# PtoeIF5A1: A Pleiotropic Regulator of Development, PCD, and Salt Tolerance in *Populus tomentosa*

**DOI:** 10.3390/plants15172702

**Published:** 2026-09-02

**Authors:** Dan Zhu, Yang Guan, Feng Feng, Lijiao Zhang, Wancong Yu, Liping Ding, Lin Zheng, Qingkuo Lan, Jianhua Wei, Yajuan Chen, Jiewei Zhang

**Affiliations:** 1 School of Environmental Engineering, Yellow River Conservancy Technical University, Kaifeng 475004, China; zhudan@yrctu.edu.cn (D.Z.);; 2 Beijing Key Laboratory of Agricultural Genetic Resources and Biotechnology, Beijing Key Laboratory of Crop Molecular Design and Intelligent Breeding, Beijing Academy of Agriculture and Forestry Sciences, Beijing 100097, China; sdqdgy211@163.com (Y.G.); weijianhua@baafs.net.cn (J.W.); 3 Institute of Germplasm Resources and Biotechnology, Tianjin Academy of Agricultural Sciences, Tianjin 300384, China

**Keywords:** *Populus tomentosa*, *PtoeIF5A1*, eukaryotic translation initiation factor 5A protein, developmental regulation, programmed cell death, salt tolerance

## Abstract

Eukaryotic translation initiation factor 5A (eIF5A) is a highly conserved protein family unique to eukaryotes, yet its functional characterization in woody plants remains limited. In this study, we identified four *eIF5A* genes (*PtoeIF5A1*–*PtoeIF5A4*) from the genome of *Populus tomentosa*, a fast-growing tree species indigenous to China, and characterized their expression patterns and functional roles through bioinformatics analysis, quantitative real-time PCR, stable overexpression in *Arabidopsis thaliana*, and transient expression in *Nicotiana benthamiana* leaves. Our results demonstrated that all PtoeIF5A proteins contain a conserved OB-fold domain and multiple phosphorylation sites, with PtoeIF5A1 showing predominant expression in roots and secondary xylem. Functional assays revealed that PtoeIF5A1 overexpression accelerated inflorescence stem elongation and early flowering in *Arabidopsis*, induced visible chlorosis and programmed cell death (PCD) in tobacco leaves, and significantly enhanced salt tolerance under NaCl treatment. Collectively, these findings establish PtoeIF5A1 in poplar as a pleiotropic regulator integrating developmental cues, programmed cell death, and stress responses; and as a valuable genetic resource for breeding stress-resilient woody plants.

## 1. Introduction

Eukaryotic translation initiation factor 5A (eIF5A, formally named eIF-4D) is a kind of small protein (16–18 kDa), which is highly conserved and common in all kinds of eukaryote organisms [1,2,3]. So far, eIF5A is the only protein contains the post translationally synthesized amino acid hypusine [Nε-(4-amino-2-hydroxyb-nutyl)lysine], which is required for its activity [4,5,6,7]. There are two enzymatic steps in hypusination. Firstly, due to deoxyhypusine synthase (DHS), a 4-amino-butyl group from sermidine is linked to the lysine of eIF5A. Secondly, 4-amino-butyl group is hydroxylated and catalyzed by deoxyhypusine hydroxylase. eIF5A is not active without hypusine [8]. Thus, hypusination is a critical regulatory point for eIF5A function.

In yeast and animals, eIF5A has been extensively characterized as a key factor in translation elongation, interacting with the 80S ribosome complex and elongation factor eEF2 [9,10,11,12,13]. Although best known for its canonical role in translation, eIF5A has also been implicated in mRNA nuclear export, cell proliferation, and programmed cell death [14,15,16,17], Its precise role in nucleocytoplasmic transport, however, remains controversial [18,19,20,21,22]. eIF5A possesses RNA-binding capacity and features a three-dimensional structure comprising two independent folded domains: an N-terminal SH3-like barrel and a C-terminal OB-fold, the latter commonly found in nucleic acid-binding proteins. Despite these advances, the precise molecular mechanisms underlying its diverse functions remain to be fully elucidated. In plants, eIF5A is encoded by a multigene family, with distinct members exhibiting divergent expression patterns and functions. In *Arabidopsis*, three eIF5A isoforms have been identified: *AteIF5A1* is highly expressed in senescent and vascular tissues, and its overexpression causes xylem enlargement, suggesting a role in lignification [23,24]; *AteIF5A2* is predominantly induced by wounding; and the *eif5a2* mutant displays aberrant morphology and enhanced resistance to apoptosis induced by fumonisin B1 or dark treatment [25]. Moreover, *AteIF5A2* has been shown to regulate programmed cell death triggered by infection with virulent *Pseudomonas syringae* pv. *tomato* DC3000 [26]. *AteIF5A3* is most abundantly expressed in seeds where active cell division occurs, and it influences growth as well as responses to osmotic and nutrient stress [27]. In addition to *Arabidopsis*, abundant *eIF5A* transcripts have been detected in senescent leaves, flowers, ripening fruits, and leaves undergoing programmed cell death in tomato [28,29], while in rice, *eIF5A* has been implicated in growth and environmental responses [30]. Collectively, these findings establish a general consensus that eIF5A plays important roles in plant senescence and stress responses. However, functional characterization of this family in woody perennial species remains entirely lacking, which prompted the present investigation.

Although the eIF5A family has been extensively studied in herbaceous model plants such as *Arabidopsis* and rice, where it is known to play critical roles in stress responses and programmed cell death, the functional characterization of *eIF5A* genes in woody plants remains largely unexplored. In particular, no study has yet investigated the structural features, post-translational modifications, or stress-related functions of eIF5A proteins in *Populus* species. This knowledge gap limits our understanding of stress resistance mechanisms in woody plants and hinders the development of molecular breeding strategies for forest trees. To address this gap, the present study aimed to isolate and characterize the *eIF5A* gene family in Chinese white poplar (*Populus tomentosa*), a commercially important woody plant. *P. tomentosa* has originated from China and has over 2000 years of cultivation history. It is an important ornamental, industrial, and fast-growing tree species in China. The first tree genome, black cottonwood (*P. trichocarpa*), has been completely sequenced and publicly released in 2006 [31]. In this study, we identified four *PtoeIF5A* genes from *P. tomentosa*. Multiple sequence alignment and motif analysis using the MEME tool revealed that all PtoeIF5A proteins contain a conserved OB-fold domain (PF01287), which is typically associated with nucleic acid binding. Prediction of post-translational modifications indicated the presence of multiple putative phosphorylation sites within these proteins. Functional characterization in transgenic *Arabidopsis* showed that overexpression of *PtoeIF5A1* confers enhanced tolerance to salt tolerance. These results provide the first evidence of the molecular mechanism underlying eIF5A1 function in woody plants, offering a theoretical basis for improving salt tolerance in forest trees.

## 2. Results

### 2.1. Bioinformatics Analysis of the eIF5A Gene Family in P. tomentosa

Exploration of the *P. tomentosa* genome through various strategies led to the identification of four genes encoding *eIF5A*, designated as *PtoeIF5A1* to *PtoeIF5A4*. PCR amplification was conducted using cDNA reverse-transcribed from total RNA extracted from *P. tomentosa* at the pre-jointing stage, resulting in four distinct bands. Sequencing analysis confirmed the presence of four unique genes, with open reading frame (ORF) lengths of 480 bp, 483 bp, 480 bp, and 483 bp, which encode deduced proteins of 160, 161, 160, and 161 amino acids, respectively. The predicted isoelectric points (pIs) of the PtoeIF5A proteins varied from 5.21 to 5.23. Furthermore, the instability indices ranged from 36.97 to 43.6, the aliphatic indices fell between 26.67 and 28.16, and all PtoeIF5A proteins were predicted to possess hydrophilic properties (Table 1).

**Figure 1:**
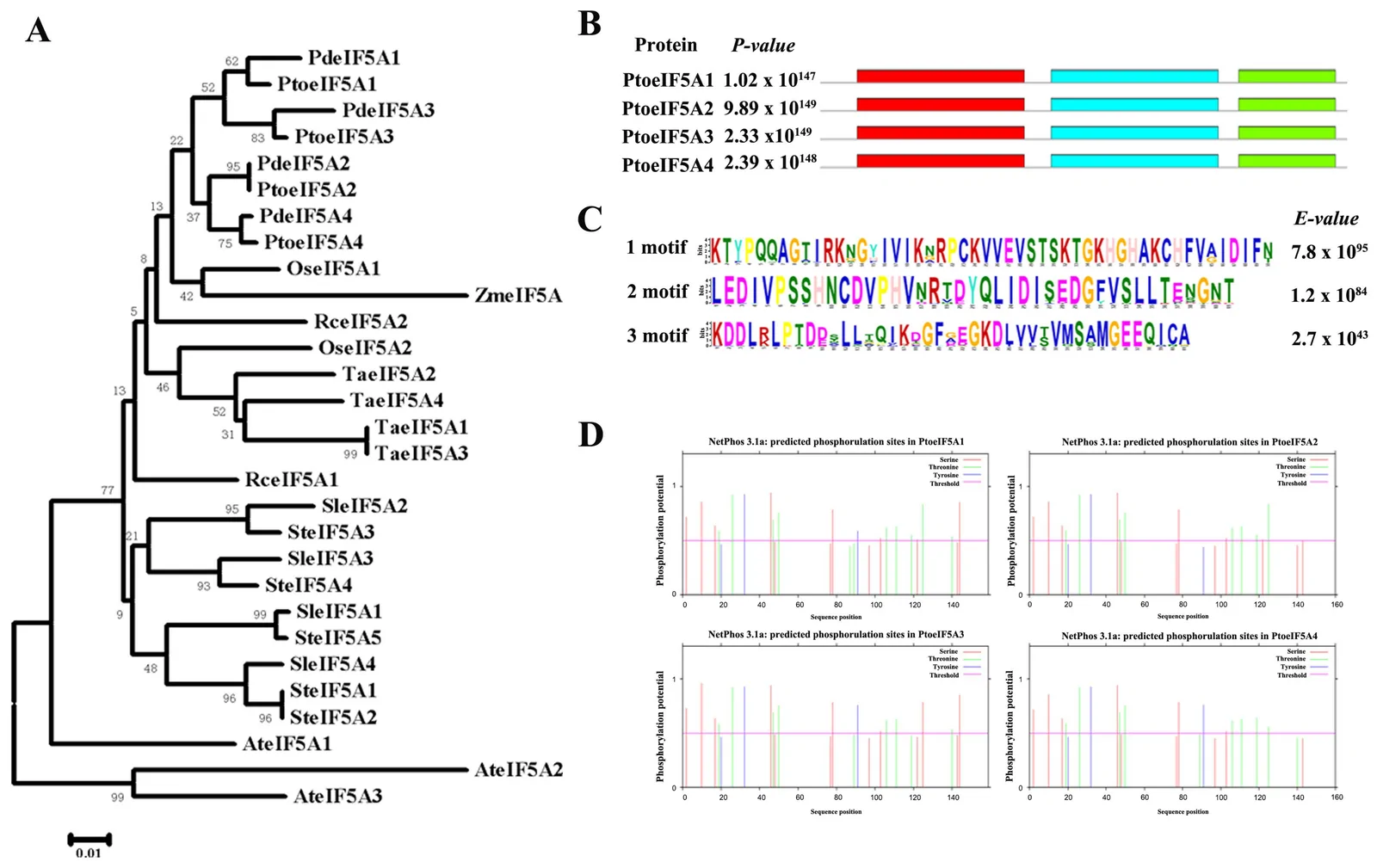
Sequence and structure analysis of the PtoeIF5A family. (**A**) Phylogenetic tree for eIF5A proteins in various plants. Pd: *Populus deltoides*; Pto: *Populus tomentosa*; Zm: Zea mays; Os: *Oryza sativa*; Ta: *Tamarix androssowii*; Rc: *Rosa hybrida*; Sl: *Solanum lycopersicum*; St: *Solanum tuberosum*; At: *Arabidopsis thaliana*. (**B**) Distribution of conserved motifs in PtoeIF5A proteins. (**C**) Sequence alignment of representative PtoeIF5As depicts the three conserved motifs. (**D**) Phosphorylation sites of PtoeIF5A proteins. The conserved residues are highlighted with different colors: red for serine, green for threonine, purple for tyrosine, and pink for the threshold.

To further investigate the evolutionary relationships of eIF5A family members across plant lineages, we extended our analysis to include 29 eIF5A amino acid sequences from various species deposited in the NCBI database, alongside the PtoeIF5As identified in this study. A phylogenetic tree was constructed using the neighbor-joining method in MEGA 6.0 (Figure 1A). The phylogenetic analysis revealed that eIF5A proteins from monocots and dicots formed distinct clades, reflecting their evolutionary divergence. Within the Salicaceae, *P. tomentosa* and *P. trichocarpa* eIF5A members clustered into well-supported subclusters, with bootstrap values above 70% for most key nodes, indicating reliable evolutionary relationships. These results suggest that the eIF5A family is evolutionarily conserved across monocots and dicots while also showing lineage-specific diversification. *P. tomentosa* and various species of *eIF5A* gene families contain three conserved domains (Figure 1B,C). NetPhos 3.1 analysis shows that PtoeIF5A1 contains 12 serines, 3 threonines, and 11 tyrosines; PtoeIF5A2 contains 12 serines, 3 threonines, and 8 tyrosines; PtoeIF5A3 contains 13 serines, 3 threonines, and 9 tyrosines; PtoeIF5A4 contains 10 serines, 3 threonines, and 10 tyrosines; and there were numerous potential phosphorylation sites (Figure 1D).

### 2.2. Tissue-Specific Expression Analysis of PtoeIF5As in P. tomentosa

**Figure 2:**
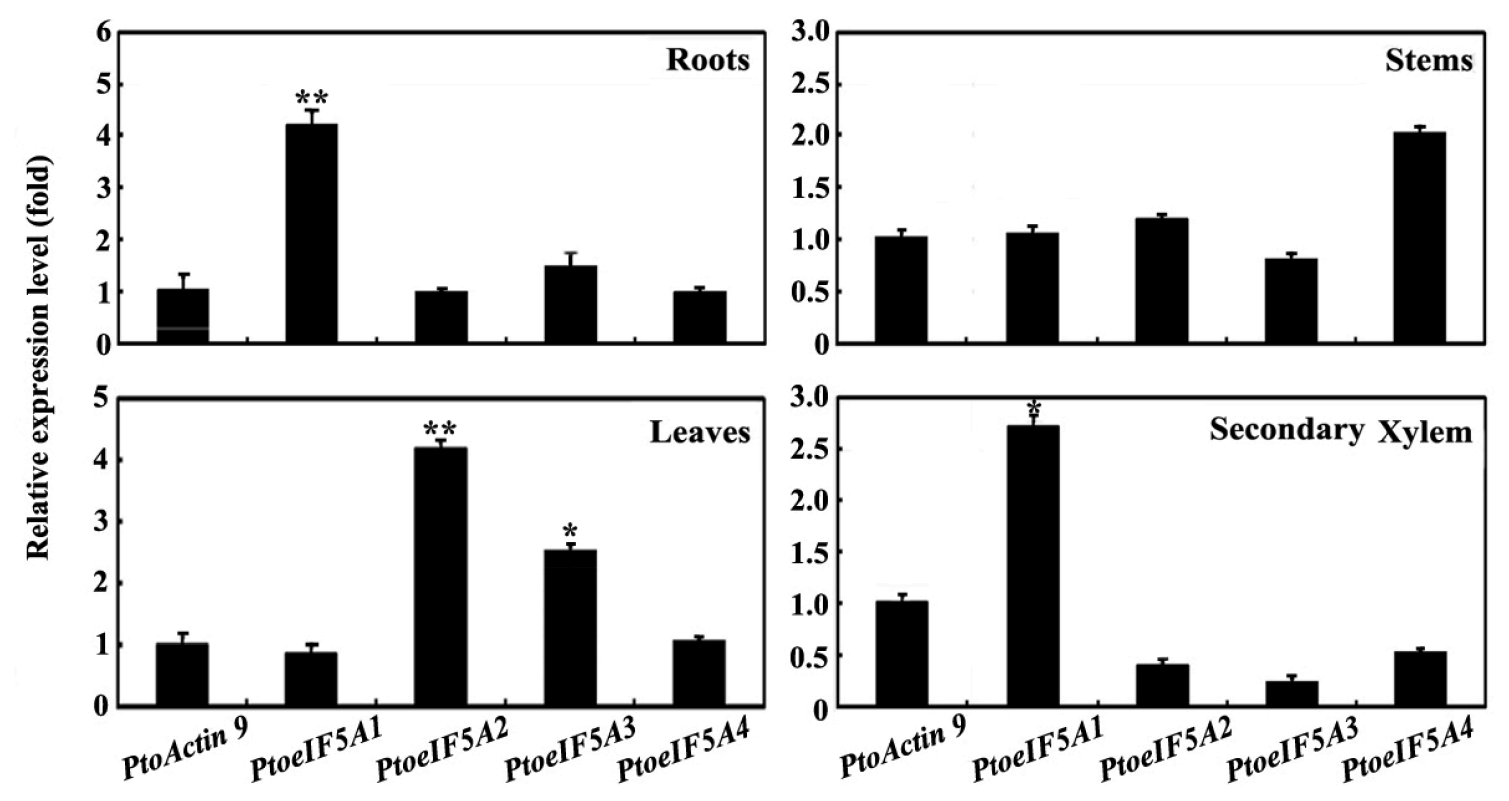
RT-qPCR analysis of *PtoeIF5A* genes expression in various organs. Gene expression was normalized to *PtoActin9* and is presented as relative expression levels relative to the control (fold change). Three independent measurements were performed. Data are presented as the mean ± SD from three replicates. Statistical significance was determined by Student’s *t*-test. Asterisk (*) indicates a difference at the *p* < 0.05 level. Asterisks (**) in each column indicate a significant difference at the *p* < 0.01 level.

The tissue-specific expression patterns of a protein are critical for understanding its biological function. To clarify the spatio-temporal expression patterns of *PtoeIF5As* in *P. tomentosa*, gene-specific primers were designed and used for quantitative real-time PCR (RT-qPCR) on cDNA derived from roots, leaves, and stems of healthy poplar plants. The results indicated that the expression patterns of the four *PtoeIF5A* family members in different organs exhibited obvious tissue specificity. In roots, the expression level of *PtoeIF5A1* was the most prominent, reaching approximately 4.2-fold, much higher than those of the other three genes, with *PtoeIF5A2* showing approximately 1.1-fold, *PtoeIF5A3* approximately 1.6-fold, and *PtoeIF5A4* approximately 1.1-fold. In secondary xylems, the expression pattern was similar to that in roots, with *PtoeIF5A1* again showing the highest level at approximately 2.7-fold, while the other three members had comparably low expression levels, approximately 0.4-, 0.2-, and 0.5-fold, respectively. In leaves, *PtoeIF5A2* showed high transcript abundance at 4.4-fold, followed by *PtoeIF5A3* at 2.6-fold, whereas *PtoeIF5A1* and *PtoeIF5A4* had comparable levels of approximately 1.0-fold. In contrast, the transcript abundance of all genes in stems was relatively low, with *PtoeIF5A4* showing the highest level at approximately 1.7-fold, and the other three at 1.1-, 1.2-, and 0.8-fold, respectively. The expression profiling described above revealed that *PtoeIF5A1* exhibited exceptionally high transcript abundance in xylem and roots, with expression levels being significantly higher than those in the other tissues examined (Figure 2). The secondary xylem serves as the primary tissue for water transport and mechanical support in plants, and its development involves complex processes including cell differentiation, secondary wall deposition, and programmed cell death. Therefore, the specific high expression of a gene in this tissue often suggests its potential involvement in regulating these critical developmental events. This expression pattern thus made *PtoeIF5A1* a candidate gene of particular interest to us.

### 2.3. Overexpression of PtoeIF5A1 Accelerates Growth in A. thaliana

**Figure 3:**
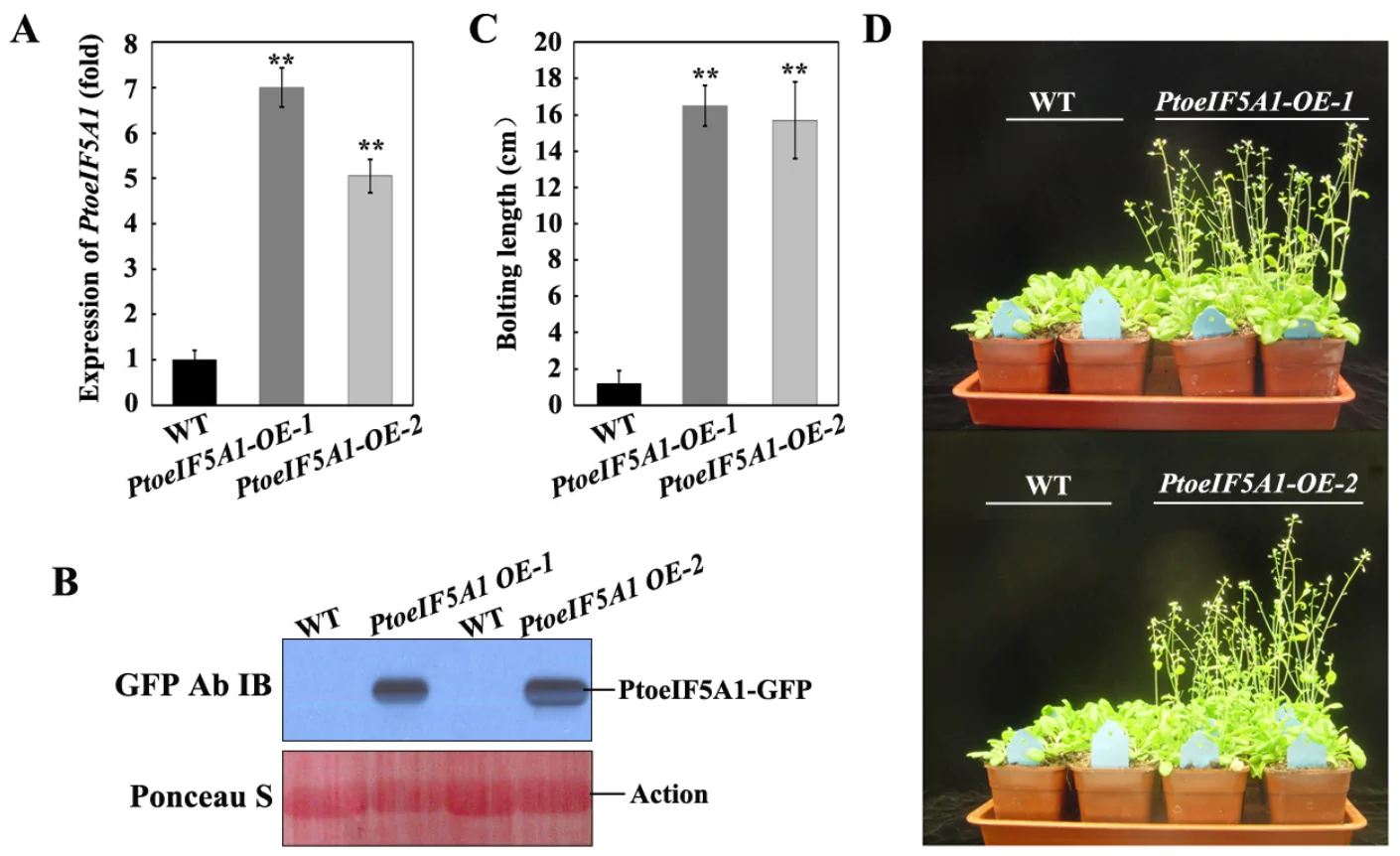
Overexpression of *PtoeIF5A1* promotes early flowering and accelerates bolting in *Arabidopsis*. (**A**) RT-qPCR analysis of the *PtoeIF5A1* transgenic *Arabidopsis* plants (OE-1 and OE-2) and WT. (**B**) Protein detection of *PtoeIF5A1* overexpression and WT seedlings. (**C**) Analysis stem length of the seedlings in (**D**). Three independent measurements were performed. Data are presented as the mean ± SD from three replicates. Statistical significance was determined by Student’s *t*-test. Asterisks (**) in each column indicate a significant difference at the *p* < 0.01 level. (**D**) Phenotypic comparison between WT and *PtoeIF5A1* overexpressing transgenic Arabidopsis lines at 8 weeks after sowing.

To investigate the molecular regulatory mechanism of PtoeIF5A1 in plants, we generated transgenic *Arabidopsis* lines overexpressing *PtoeIF5A1* under the control of the cauliflower mosaic virus 35S promoter via *Agrobacterium tumefaciens*-mediated transformation. Homozygous T_2_ generation transgenic lines were obtained following hygromycin resistance selection and confirmed by genomic PCR. Two independent overexpression lines, designated OE-1 and OE-2, were selected for further characterization. RT-qPCR analysis revealed that *PtoeIF5A1* transcript levels were approximately 7-fold and 5-fold higher in OE-1 and OE-2, respectively, compared with wild-type (WT) controls, confirming successful overexpression of the transgene at the transcriptional level (Figure 3A). To verify the translation of the PtoeIF5A1 protein, total proteins extracted from transgenic and WT plants were subjected to Western blot analysis using an anti-GFP antibody, as the overexpression construct encodes a PtoeIF5A1–GFP fusion protein. A specific band of approximately 45 kDa, corresponding to the predicted molecular mass of the PtoeIF5A1-GFP fusion protein, was detected in the transgenic lines but not in the WT controls (Figure 3B). These results collectively confirm that the *PtoeIF5A1* transgene is both transcribed and properly translated into the full-length fusion protein in transgenic *Arabidopsis*, providing a solid basis for subsequent subcellular localization and functional assays.

To investigate the effect of *PtoeIF5A1* overexpression on plant developmental progression, overexpression of *PtoeIF5A1* plant OE-1 and OE-2, with WT as controls, were cultivated under identical conditions and subjected to phenotypic comparison at 8 weeks of age. The results showed that *PtoeIF5A1* overexpression significantly promoted plant growth and development, with the most prominent phenotypic manifestation being earlier bolting initiation and accelerated inflorescence stem elongation (Figure 3D). At 8 weeks, the mean bolting stem lengths of OE-1 and OE-2 lines reached 16.5 cm and 15.8 cm, respectively, whereas WT plants had just initiated bolting, with a mean bolting stem length of only 1.1 cm (Figure 3C). In addition, flowering time was substantially advanced in the *PtoeIF5A1* overexpression lines, as evidenced by a significant reduction in both the days to flowering and the rosette leaf number at the flowering stage compared with the WT control (Figure S2). These findings indicate that heterologous overexpression of *PtoeIF5A1* substantially accelerates inflorescence stem elongation, thereby significantly advancing the vegetative-to-reproductive transition, namely resulting in a distinct early-flowering phenotype.

### 2.4. Overexpression of PtoeIF5A1 Induces Premature Senescence in Tobacco Leaves

**Figure 4:**
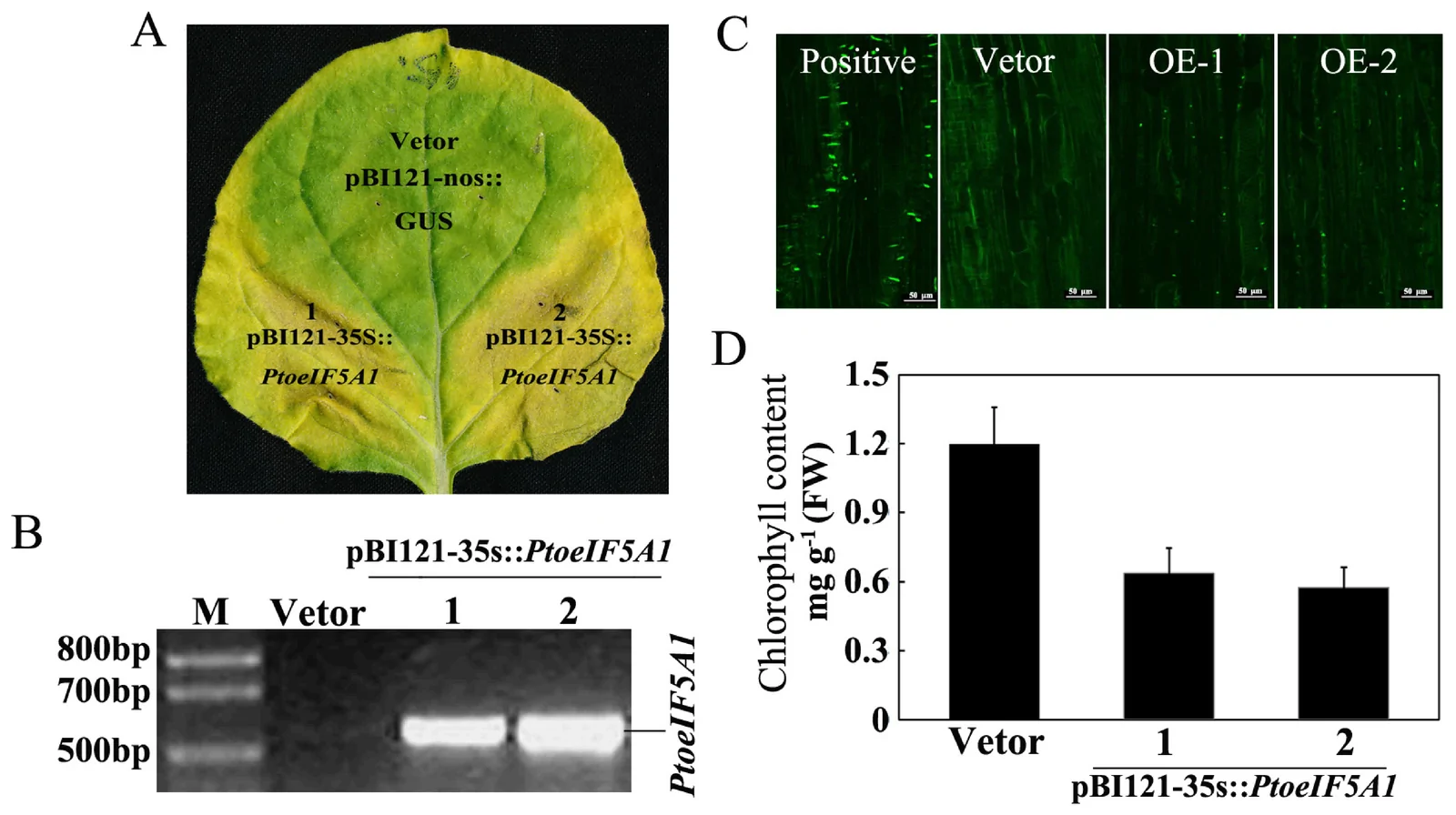
Transient overexpression of *PtoeIF5A1* induces programmed cell death and premature leaf senescence in *N. benthamiana.* (**A**) Phenotypes of tobacco leaves at 7 days post-infiltration (dpi) with *Agrobacterium* carrying the empty vector control (pBI121-nos::GUS) or the constitutive expression construct pBI121-35S∷PtoeIF5A1. (**B**) PCR confirmation of transient transformation. (**C**) TUNEL staining of infiltrated leaf tissues. The scale bar = 50 µm. (**D**) Chlorophyll a and chlorophyll b contents in the indicated leaves. Three independent measurements were performed. Data are presented as the mean ± SD from three replicates. Statistical significance was determined by Student’s *t*-test.

Given that the aforementioned results indicated that overexpression of *PtoeIF5A1* accelerates developmental progression in *Arabidopsis*, we further investigated whether this gene is involved in the regulation of programmed cell death (PCD). To this end, we employed an *Agrobacterium*-mediated transient transformation system, in which a construct carrying *PtoeIF5A1* was infiltrated into leaves of *Nicotiana benthamiana* via syringe injection to achieve transient high-level expression of the transgene in leaf cells (Figure 4A). Two treatment groups were established: the empty vector control pBI121-nos::GUS and the constitutive expression construct pBI121-35S::PtoeIF5A1. To verify the efficiency of transient transformation, total DNA was extracted from infiltrated leaves and subjected to PCR analysis. The specific transgene band was amplified exclusively in PtoeIF5A1-infiltrated leaves, confirming successful transient integration and expression of the transgene (Figure 4B). At 7 days post-infiltration, leaves transiently expressing *PtoeIF5A1* exhibited obvious chlorosis, whereas no visible yellowing was observed in either the empty vector control or non-infiltrated leaves, suggesting that this gene may induce leaf senescence. To further ascertain whether the chlorosis resulted from PCD, we performed TUNEL staining on the yellowing leaves, which revealed clear PCD-specific signals (Figure 4C). Concurrently, to quantify the degree of chlorosis and evaluate chloroplast integrity, we measured the chlorophyll content in each treatment group. The results showed that, compared with the controls, the chlorophyll a and chlorophyll b contents in *PtoeIF5A1*-expressing leaves were significantly reduced, with decreases of approximately 45% and 50%, respectively (Figure 4D). Collectively, based on molecular and physiological evidence, we preliminarily infer that transient overexpression of *PtoeIF5A1* in tobacco leaves may promote the translation of PCD-related proteins, thereby interfering with normal photosynthetic metabolism, leading to chlorophyll degradation, and ultimately triggering PCD.

### 2.5. Overexpression of PtoeIF5A1 Increases Salt Tolerance in A. thaliana

It is noteworthy that previous studies have shown that eIF5A family members are not only involved in vascular tissue development but also broadly participate in stress responses in various plant species. Given the known functions of its family members in abiotic stress responses, we aimed to further investigate the expression changes of *PtoeIF5A1* under stress conditions including salt, drought, high light, low temperature, and alkaline stress, in order to evaluate its potential role in plant stress adaptation.

**Figure 5:**
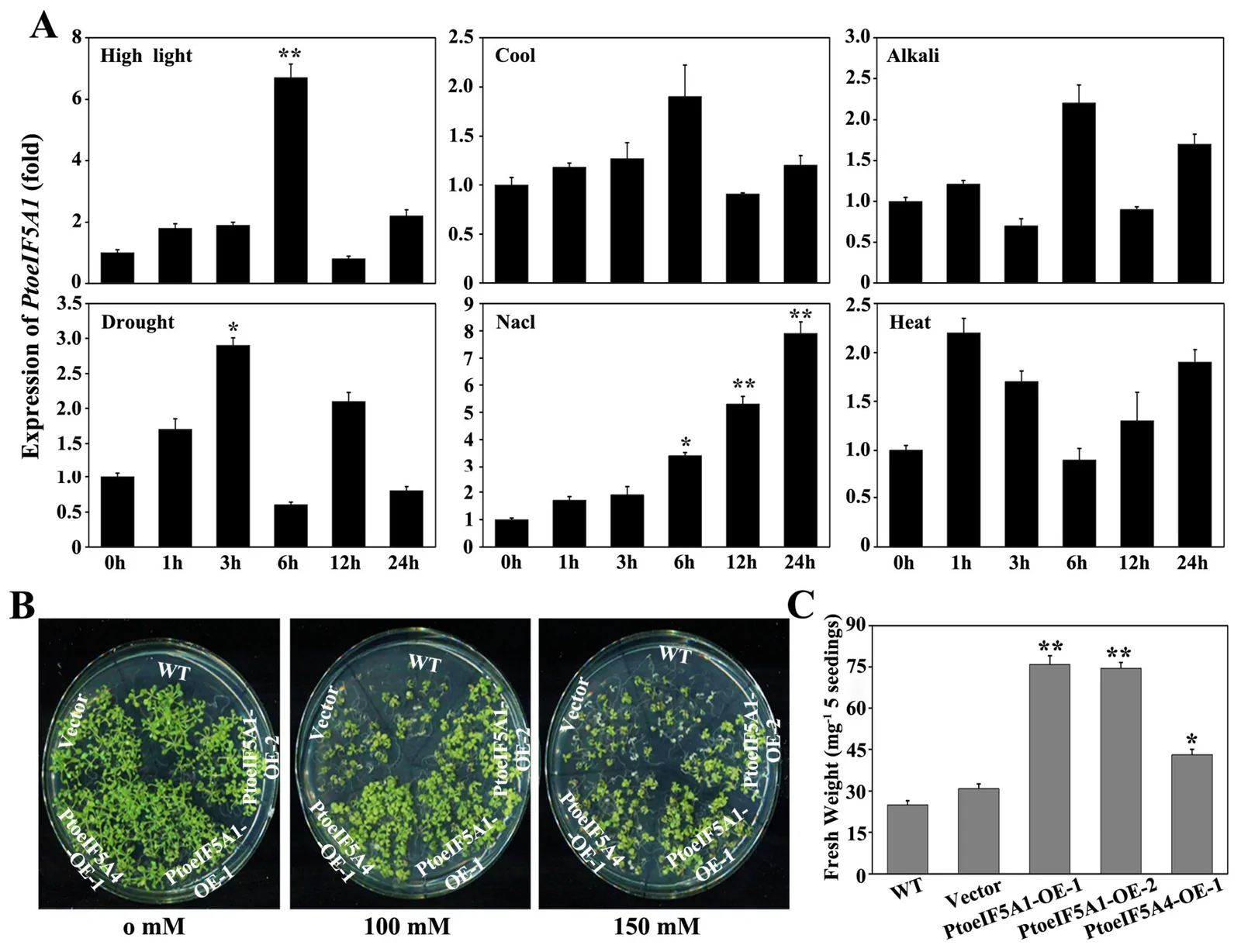
Overexpression of the *PtoeIF5A1* gene in *Arabidopsis* enhances salt tolerance resistance. (**A**) *PtoeIF5A1* gene expression under 1200 μMm^−2^s^−1^, drought, 150 mM NaCl, 550 mM KOH, 4 °C, and 42 °C. The *PtoActin9* samples were used as an internal reference. In response to the various stress treatments, roots were specifically selected for analysis. All assays were repeated three times, and all values were the means of three replicates with 10 plants. Data are presented as the mean ± SD from three replicates. Statistical significance was determined by Student’s *t*-test. Asterisk (*) indicates a difference at the *p* < 0.05 level. Asterisks (**) in each column indicate a significant difference at the *p* < 0.01 level. (**B**) WT, two independent *PtoeIF5A1* overexpression lines (OE-1 and OE-2), and *PtoeIF5A4* transgenic seedlings were grown on MS medium supplemented with 0, 100, or 150 mM NaCl for 2 weeks, after which representative photographs were taken. (**C**) Analysis of the fresh weight of the seedlings in (**B**). Three independent measurements were performed. Data are presented as the mean ± SD from three replicates. Asterisk (*) indicates a difference at the *p* < 0.05 level. Asterisks (**) in each column indicate a significant difference at the *p* < 0.01 level.

In order to examine the effects of various abiotic stresses on the expression level of *PtoeIF5A1*, we performed RT-qPCR analysis of total RNA prepared from leaves of trees (Figure 5A). The results showed that *PtoeIF5A1* expression was significantly induced by high light, low temperature, salt, heat, drought, and alkaline stress, with varying degrees of upregulation observed as early as 1 h after all treatments, suggesting that this gene can rapidly respond to diverse stress signals. Further analysis revealed that *PtoeIF5A1* exhibited distinct temporal expression patterns under different stress conditions: its expression peaked at 1 h under heat stress, at 3 h under drought stress, and at 6 h under high light, low temperature, and alkaline stress. Notably, under the aforementioned stresses, *PtoeIF5A1* expression declined to varying extents after reaching the peak. In striking contrast, under salt stress, *PtoeIF5A1* was strongly induced and upregulated starting from 1 h post-treatment, and its transcript levels continuously accumulated throughout the treatment period, showing an overall upward trend over time without any apparent decline, indicating that *PtoeIF5A1* exhibits a sustained and stable response to salt stress.

To determine the function of *PtoeIF5A1* in the salt stress response of seedlings, we analyzed the growth phenotypes of transgenic *Arabidopsis* seedlings overexpressing *PtoeIF5A1* under different concentrations of NaCl stress. As shown in Figure 5B, in the absence of NaCl treatment, there was no difference in growth between the transgenic plants and WT controls. However, upon NaCl treatment, all plants displayed a salt tolerance-sensitive phenotype characterized by smaller aerial parts and shorter primary roots. Compared with WT, the *PtoeIF5A1* transgenic plants showed stronger salt tolerance. we measured the fresh weight of the aerial parts of seedlings grown on MS medium containing 150 mM NaCl (Figure 5C). It was found that with the increasing NaCl concentration in MS, the average fresh weight of the two *PtoeIF5A1* transgenic lines was 44.28%, and 39.3% higher than that of the WT, respectively.

To further evaluate the physiological basis of salt tolerance, we measured relative ion leakage (RIL) and free proline accumulation in *Arabidopsis* leaves under salt tolerance conditions. As shown in Figure S1A, salt tolerance caused a significant increase in RIL in all genotypes relative to controls, indicating substantial membrane damage; however, the *PtoeIF5A1* transgenic plants exhibited lower RIL than *PtoeIF5A4* transgenic plants and WT under salt tolerance, reflecting superior membrane integrity. Concurrently, proline as a key osmoprotectant accumulated to significantly higher levels in *PtoeIF5A1* transgenic plants than in *PtoeIF5A4* transgenic plants and WT under salt tolerance, implying that enhanced osmotic adjustment contributes to cellular protection in the overexpressing lines (Figure S1B). These results indicate that overexpression of *PtoeIF5A1* enhances salt tolerance in transgenic seedlings.

## 3. Materials and Methods

### 3.1. Plant Materials and Treatment

To test tissue-specific expression, three-year-old white poplar plants (*P. tomentosa* cv. ‘BJHR01’) were examined. These plants were grown in an experimental orchard at the Beijing Academy of Agriculture and Forestry Sciences (Beijing, China). Their secondary xylem (1.5 m height from land), newly developed lateral roots (1–2 mm diam.), and leaves sampled from the middle part of new shoots were collected on 5 April 2013. The in vitro vegetative propagation of white poplar was routinely carried out using established techniques [32]. Six-month-old plantlets of *P. tomentosa* were grown in a greenhouse at 25 °C under a 16 h light/8 h dark cycle at the Beijing Academy of Agriculture and Forestry Sciences, China. Cold (4 °C), drought, salt (150 mM NaCl), high light (1200 μM m^−2^s^−1^), alkali (550 mM KOH), and heat (42 °C) treatments were performed as described formerly [33], and the sampling time points were 0, 3, 6, 12, and 24 h for gene expression analysis. For all treatments, leaves were collected at various time points and immediately frozen with liquid nitrogen and stored in a refrigerator at −80 °C until RNA extraction.

Seeds of *Arabidopsis* (Col-0) were surface-sterilized and germinated on Petri dishes containing half-strength MS medium (Murashige and Skoog basal medium, Sigma-Aldrich, St. Louis, MO, USA). After germination, seedlings were transferred to MS medium supplemented with 0, 100, or 150 mM NaCl (Sigma-Aldrich, St. Louis, MO, USA) and grown in a growth room under a photoperiod of 14 h light/10 h dark (100 μmol m^−2^ s^−1^), with day/night temperatures of 23 °C/20 °C and a relative humidity of 70%. Seedlings were cultured for two weeks under these conditions prior to harvest for phenotypic and physiological analyses.

### 3.2. Isolation and Sequence Analysis of PtoeIF5As

Since *P. tomentosa* and *P. trichocarpa* are closely related homologous species, and the genome of *P. trichocarpa* has been finely sequenced and annotated with high genomic similarity to *P. tomentosa*, the bioinformatics analyses of *eIF5As* in this study were performed using the *P. trichocarpa* genome. Through BLAST (2.13.0) homology searches and conserved domain validation using MEME (5.5.2), we identified four *eIF5A* genes in the *P. trichocarpa* genome, designated as *PtreIF5A1-PtreIF5A4*. Based on the identified *P. trichocarpa* sequences, gene-specific primers were designed for cloning the corresponding homologs from *P. tomentosa*.

Total RNA was extracted from leaf samples of *P. tomentosa* using the Plant RNAout kit (TIANGEN, Beijing China). One microgram of total RNA was treated with DNase I (Promega, Madison, WI, USA) according to the manufacturer’s instructions. First-strand cDNA was synthesized using the SuperScript™ RT Reagent Kit (Takara, Dalian, China). The full-length cDNAs of *PtoeIF5A* genes were then amplified by homologous cloning using the designed primers (Supplementary Table S1). Nested PCR was performed to obtain *PtoeIF5As* (GenBank accession number HQ529479), and the PCR products were visualized on a 1.5% agarose gel. The target fragments were purified using the Gel Extraction Kit (Promega, Corporation, Madison, WI, USA), TA-ligated into the pGEM-T-easy Vector (Promega, Corporation, Madison, WI, USA), and sequenced.

### 3.3. Bioinformatics Analysis of PtoeIF5As

The deduced amino acid sequences of PtoeIF5As, together with those of other reported plant eIF5As, were aligned using ClustalX 1.83. A phylogenetic tree was constructed using the neighbor-joining method in MEGA 6.0, with bootstrap support assessed from 1000 replicates to confirm their classification and integrity. The gene IDs of all species used for phylogenetic tree construction are listed in Supplementary Table S2. We identified conserved domains within the PtoeIF5As protein sequence using the Pfam database v34.0 (http://pfam.xfam.org/).

We conducted a comprehensive protein analysis employing various online tools: conserved domains within the PtoeIF5A protein sequences were identified using the Pfam database (http://pfam.xfam.org/). Comprehensive protein analyses were performed using various online tools: phosphorylation sites were predicted with NetPhos 3.1 (https://services.healthtech.dtu.dk/service.php?NetPhos-3.1 (accessed on 23 August 2026)), hydrophobicity was assessed using ProtScale (http://web.expasy.org/protscale/ (accessed on 23 August 2026)), transmembrane domains were predicted with TMHMM (https://services.healthtech.dtu.dk/service.php?TMHMM-2.0) (accessed on 23 August 2026)), signal peptides were identified using SignalP 5.0 (https://services.healthtech.dtu.dk/service.php?SignalP-5.0 (accessed on 23 August 2026)), secondary structure was analyzed with SOPMA (http://npsa-pbil.ibcp.fr/cgi-bin/npsa_automat.pl?page=npsa_sopma.html (accessed on 23 August 2026)), and tertiary structure was predicted using Swiss-Model (http://beta.swissmodel.expasy.org).

### 3.4. Quantitative RT-PCR

To test *PtoeIF5A1* expression under various abiotic stresses, the sixth leaf position (LP6, leaf position 6) of 6 months *P. tomentosa* was generally used. Total RNA was extracted and purified as described above. DNA-free total RNA was reverse-transcribed into cDNA using the SuperScript^TM^ III Reverse Transcriptase kit (Invitrogen, Carlsbad, CA, USA). Gene-specific primers were designed with Primer3 Plus 3.2.0 and are listed in Supplementary Table S1. The *PtoAction9* gene was used as the reference to normalize gene expression across the samples. RT-qPCR was performed in a total volume of 15 μL containing 1 μL of cDNA, 1.5 μL of each gene specific primer (0.5 μM), and 5 μL of Premix EX TaqTM (Takara, Dalian, China). RT-qPCR was performed in duplicate in a StepOnePlus™ RT-qPCR System (Applied Biosystems, Foster City, CA, USA) using the following program: 95 °C for 30 s for initial denaturation; and 40 cycles of 95 °C for 5 s, 60 °C for 35 s. Each RT-qPCR experiment was performed with three independent biological replicates.

### 3.5. Generation of Transgenic Arabidopsis Plants

To obtain the overexpression construct, the full-length *PtoeIF5A1* gene was cloned into a modified pBluescript vector with *Nde* I/*Spe* I sites. The *PtoeIF5A1* with GFP tag coding sequence was inserted into a modified pBI121 vector with *Xho* I/*Spe* I sites and then electroporated into *A. tumefaciens* strain GV3101, which was subsequently used for the transformation of *Arabidopsis*.

Transgenic plantlets were selected on 0.5 × MS agar medium (1% agar *w*/*v*, BD, Franklin Lakes, NJ, USA) supplemented with kanamycin (50 mg/L). T1 transgenic lines were allowed to self-fertilize, and transgenic seeds were selected based on 3:1 segregation for kanamycin resistance in the T_2_ generation. T_3_ homozygous lines were derived and analyzed by Western blotting. A total of eight independent transgenic lines were generated, among which seven lines exhibited detectable expression of the target protein, corresponding to a transformation efficiency of approximately 90%. Two independent lines (OE-1 and OE-2) with relatively higher PtoeIF5A1 protein accumulation were selected for subsequent phenotypic and physiological analyses.

### 3.6. Protein Extraction and Immunoblot Analysis

Total proteins were extracted from leaf tissue by grinding with a small plastic pestle in extraction buffer. After centrifugation at 15,000 rpm for 30 min, supernatants were transferred into other tubes. The concentration of protein extracts was determined using the Bio-Rad protein assay kit (Bio-Rad, Hercules, CA, USA) with bovine serum albumin as the standard. For the immunoblot analysis, 10 μg of total proteins per lane were separated by electrophoresis on 12% sodium dodecyl sulfate (SDS)–polyacrylamide gels [34], and then the proteins were transferred to nitrocellulose membranes (Millipore, Billerica, MA, USA). Equal protein loading was verified by Ponceau S staining of the blotted membrane prior to immunodetection. After blocking with defatted milk, the blot was incubated with primary antibody (anti-GFP, 10,000-fold dilution) and then with horseradish peroxidase-conjugated goat anti-mouse IgG (10,000-fold dilution). The blot was incubated with Lumi-light substrate (Roche, Basel, Germany) according to the manufacturer’s instructions.

### 3.7. TUNEL Assay for Detection of Programmed Cell Death

Leaf samples from treated and control plants were fixed, dehydrated through a graded ethanol series, cleared in xylene, infiltrated with paraffin, and sectioned at 30 µm thickness. After deparaffinization and rehydration, sections were treated with 20 µg/mL proteinase for 10 min at room temperature, post-fixed in 4% paraformaldehyde, and equilibrated with equilibration buffer. The TdT reaction mixture (containing nucleotide mix and terminal deoxynucleotidyl transferase) was applied and incubated at 37 °C for 60 min in the dark. The reaction was terminated with 2× SSC, followed by three washes each with PBS and deionized water. Green fluorescence, indicative of DNA fragmentation, was visualized under a confocal microscope at 520 nm. Positive controls were pre-treated with DNase I, and negative controls omitted the TdT enzyme.

### 3.8. Agrobacterium-Mediated Transient Transformation of Tobacco Leaves

The expression construct pBI121-35S::PtoeIF5A1 and the empty vector control pBI121-nos::GUS were separately introduced into *Agrobacterium tumefaciens* strain GV3101 competent cells. Positive single colonies were picked and cultured in LB liquid medium supplemented with 50 mg/L kanamycin and 25 mg/L rifampicin at 28 °C with shaking at 200 rpm until the mid-logarithmic phase (OD_600_ = 0.8). The bacterial cells were harvested by centrifugation at 4000 *g* for 10 min, resuspended in infiltration buffer (10 mM MES, 10 mM MgCl_2_, 200 µM acetosyringone, pH = 5.6) to a final OD_600_ of 0.6, and allowed to stand at room temperature for 3 h. Fully expanded leaves from 4-week-old *N. benthamiana* plants were infiltrated with the bacterial suspension using a needleless 1 mL syringe, gently pressing the suspension into the mesophyll from the abaxial side until the infiltrated zones spread across the entire lamina. At least three leaves per plant and three independent plants per construct were infiltrated. The inoculated plants were grown in a greenhouse at 22 °C under a 16 h light/8 h dark photoperiod. Leaf discs from the infiltrated areas were harvested at 3, 5, and 7 days post-infiltration for subsequent analyses.

### 3.9. Measurement of Membrane Ion Leakage and Proline Production

Ion leakage was measured using a LeiCi DDS-307A conductivity meter (INESA Scientific Instrument Co., Ltd., Shanghai, China). For each replicate, 10 seedlings from the salt-stressed treatment group were placed into a 15 mL centrifuge tube containing 10 mL of deionized water. The leaf samples were subjected to vacuum infiltration for 30 min at room temperature, followed by incubation on an orbital shaker with gentle agitation for 2 h at 25 °C. The initial conductivity of the bathing solution was measured and recorded as EC_1_. Subsequently, the tubes were heated in a water bath for 15 min to completely release total cellular electrolytes, and after cooling to room temperature, the total conductivity was recorded as EC_2_. The conductivity of deionized water without leaf samples was used as the blank control (EC_0_). The relative ion leakage (RIL) was calculated as RIL(%) = [(EC_1_ − EC_0_)/(EC_2_ − EC_0_)] × 100%. For salt-treated samples, an additional blank control containing 150 mM NaCl solution (without leaf samples) was measured to correct for background conductivity contributed by residual surface salt, and leaves were briefly rinsed with deionized water prior to measurement to remove surface-adherent NaCl crystals. All measurements were performed with three independent biological replicates, and data are presented as the means ± SD.

Fresh *Arabidopsis* leaves (0.2 g) were weighed and placed into a glass test tube, to which 5 mL of freshly prepared 3% sulfosalicylic acid was added. The mixture was incubated in a boiling water bath for 10 min to fully extract free proline. After cooling to room temperature, the extract was filtered through gauze, and the resulting filtrate was used as the crude proline extract. An aliquot of 2 mL of this extract was transferred to a stoppered glass test tube, along with 2 mL of glacial acetic acid and 3 mL of 2.5% acid ninhydrin, while double-distilled water served as the blank control; the reaction mixture was then heated in a boiling water bath for 40 min to allow complete color development. After a second cooling step, 5 mL of toluene was added and vigorously mixed to partition the chromophore into the organic phase. Upon standing at room temperature, the upper toluene layer was carefully collected after phase separation, and its absorbance was measured at 520 nm. The proline content in the sample was determined by referring to a standard curve, and the final proline concentration in the original leaf tissue was calculated using the provided formula, where X denotes the proline amount obtained from the standard curve.

### 3.10. Chlorophyll Content Determination

Chlorophyll content was measured using the acetone extraction method. Fresh leaves at the same node position from treated and control groups were collected, and leaf discs (0.5 cm in diameter) were punched out while avoiding the main veins (or approximately 0.1 g of fresh weight was weighed). The samples were cut into pieces, ground to a fine powder with liquid nitrogen in a mortar, and transferred to centrifuge tubes. Then, 5 mL of 80% acetone was added, and the mixture was incubated at 4 °C in the dark for 24 h for pigment extraction. After centrifugation at 5000 *g* for 10 min at 4 °C, the supernatant was collected. The absorbance was measured at 663 nm and 645 nm using a spectrophotometer with 80% acetone as a blank. Chlorophyll a, chlorophyll b, and total chlorophyll concentrations were calculated according to the Arnon equations. Finally, the chlorophyll content was expressed as milligrams per gram fresh weight (mg/g FW) based on the sample fresh weight.

## 4. Discussion

This study systematically identified the *eIF5A* gene family in *P. tomentosa* and investigated the roles of PtoeIF5A1 in developmental regulation, PCD, and salt tolerance. RT-qPCR analysis revealed that *PtoeIF5A1* was predominantly expressed in roots and secondary xylem (Figure 2), with much lower transcript levels in stems and leaves. Transient overexpression of *PtoeIF5A1* in tobacco leaves resulted in obvious chlorosis, reduced chlorophyll content (Figure 4D), and TUNEL-positive signals (Figure 4C), indicating the induction of PCD. In transgenic *Arabidopsis*, overexpression of *PtoeIF5A1* enhanced salt tolerance, as evidenced by increased fresh weight and reduced ion leakage under salt tolerance (Figure 5). Additionally, salt tolerance induced a persistent and robust upregulation of *PtoeIF5A1* transcript levels over a 24 h period, in contrast to the transient responses observed under other stress conditions (Figure 5A). In addition, we examined the transcriptional responses of *PtoeIF5A2*, *PtoeIF5A3*, and *PtoeIF5A4* to various abiotic stresses (Figure S3). Notably, these three family members exhibited expression patterns that were distinctly different from those of *PtoeIF5A1* under salt, drought, high temperature, low temperature, high light, and alkaline stresses. Among them, *PtoeIF5A2* was preferentially responsive to high light and high temperature and *PtoeIF5A3* showed marked sensitivity to alkaline stress, whereas *PtoeIF5A4* was significantly induced under drought and high-temperature conditions, with distinct amplitudes of induction observed among the members. This divergence in stress-responsive profiles suggests that different members of the PtoeIF5A family may assume non-redundant functional roles in response to specific environmental cues. Considering the sustained and robust upregulation of *PtoeIF5A1* under salt stress, together with its functions in PCD and vascular development, we propose that functional specialization has occurred among PtoeIF5A members during evolution; *PtoeIF5A1* tends to participate in developmental PCD and salt stress adaptation, whereas *PtoeIF5A2/3/4* may primarily mediate specific responses to drought, extreme temperatures, and alkaline stress. This expression differentiation provides important clues for further dissecting the division of labor among eIF5A family members within the multi-stress adaptation network of woody plants and highlights the need for more refined functional characterization of individual family members in response to their cognate stress types in future studies.

Further analysis of the protein structure provides a molecular explanation for the aforementioned developmental correlations. Sequence alignment revealed that all PtoeIF5A proteins contain a conserved OB-fold domain and multiple putative phosphorylation sites (Figure 1B–D). The OB-fold is commonly found in nucleic acid-binding proteins, suggesting that PtoeIF5A may regulate translation elongation by binding to specific mRNAs or ribosomal components, thereby influencing downstream gene expression. This structural characteristic appears particularly critical for PCD regulation. In the present study, transient overexpression of *PtoeIF5A1* in tobacco leaves led to obvious chlorosis, reduced chlorophyll content (Figure 4D), and TUNEL-positive signals (Figure 4C), clearly indicating the induction of PCD. This observation parallels the PCD process occurring during secondary xylem maturation, implying that PtoeIF5A1 promotes programmed cell death by translationally activating PCD-associated factors, such as cysteine proteases or nucleases. Similarly, *AteIF5A2* has been reported to regulate PCD upon pathogen infection, while *AteIF5A1* has been associated with xylem-related PCD [23,35,36]. Collectively, these findings suggest that the structural attributes of PtoeIF5A1 enable it to specifically regulate the translation of PCD-associated mRNAs, thereby playing a central role in both vascular maturation and stress-induced cell death.

Strikingly, the function of PtoeIF5A1 extends beyond developmental regulation to play a significant role in abiotic stress responses. Among the various abiotic stresses examined, salt stress induced the most persistent and robust upregulation of *PtoeIF5A1*, with transcript levels continuously increasing over a 24 h period without declining, in stark contrast to the transient responses observed under other stress conditions (Figure 5A). To assess the functional significance of this salt-induced expression, we generated *PtoeIF5A1*-overexpressing transgenic *Arabidopsis* lines. These transgenic lines exhibited enhanced salt tolerance compared with wild-type plants, as evidenced by increased fresh weight and reduced ion leakage under salt stress (Figure 5C). Collectively, these results demonstrate that PtoeIF5A1 functions as a positive regulator of salt tolerance in plants. Given the abundant expression of *PtoeIF5A1* in roots, we hypothesize that its preferential accumulation in this tissue may facilitate the sensing of soil salinity fluctuations, thereby enabling rapid activation of adaptive responses. This hypothesis is consistent with the proposed role of root-expressed genes in the early perception of salt stress. Notably, this hypothesis is further supported by independent studies in other species: *eIF5A* genes in rice have been shown to be transcriptionally regulated by salt and heavy metal stress [37,38], and eIF5A functions as a translation regulator under stress conditions in eukaryotic cells [39]. More importantly, a homolog from *Tamarix androssowii* (*TaeIF5A1*) has been demonstrated to enhance abiotic stress tolerance when overexpressed in transgenic poplar, with transgenic plants exhibiting lower electrolyte leakage and higher chlorophyll content under salt stress [40]. These findings collectively support a conserved role for eIF5A in stress tolerance across diverse plant species. However, direct evidence for the involvement of PtoeIF5A1 in salt sensing or signal transduction specifically in roots remains to be obtained. Future studies employing root-specific promoters to drive *PtoeIF5A1* expression or tissue-specific knockdown approaches could be instrumental in testing this hypothesis.

At the protein structural level, the conserved phosphorylation sites identified in PtoeIF5A1 raise the possibility that these residues may serve as modification targets for stress-related signaling pathways, potentially including the MAPK cascade, and that such phosphorylation events may modulate its translational activity or substrate selectivity. This hypothesis is based on the well-established role of phosphorylation in regulating protein function [38,41,42,43,44] and the presence of multiple predicted phosphorylation sites in PtoeIF5A1. However, it is important to note that no experimental evidence currently links PtoeIF5A1 to MAPK signaling or other phosphorylation-dependent regulatory networks in Populus. Phosphorylation assays, such as in vitro kinase assays or phospho-specific antibody-based detection, combined with site-directed mutagenesis of the predicted phosphorylation sites, are needed to determine whether these modifications occur and whether they are functionally relevant. Furthermore, in light of the pleiotropic nature of eIF5A as a translation elongation factor and the observation that PtoeIF5A1 is strongly induced by salt stress (Figure 5A), we propose that the pronounced accumulation of PtoeIF5A1 in secondary xylems may reflect its broader involvement in stress-associated developmental programs. This hypothesis is supported by our finding that transient overexpression of PtoeIF5A1 in tobacco leaves elicits a premature senescence phenotype, suggesting a functional link between stress responses and developmental PCD. Nevertheless, the molecular connection between stress-induced senescence and xylem differentiation in the context of PtoeIF5A1 function remains speculative. Future research integrating transcriptomic, metabolomic, and physiological analyses in PtoeIF5A1-overexpressing and knockdown lines will be essential to clarify the mechanistic relationships among stress signaling, developmental PCD, and translational control mediated by PtoeIF5A1.

Together with established links between eIF5A and plant senescence in model species such as *Arabidopsis*, these lines of evidence support the hypothesis that PtoeIF5A1 functions as a molecular hub that couples developmental signals, environmental stress cues, and senescence responses at the level of translational regulation. However, the current conclusions are primarily derived from gain-of-function approaches, and loss-of-function evidence remains unavailable. Future investigations should prioritize the generation of *PtoeIF5A1* loss-of-function mutants in *Populus* using RNAi knockdown or CRISPR/Cas9-mediated mutagenesis to further substantiate its endogenous functions in development, PCD, and salt tolerance responses. In parallel, genome-wide identification of direct translational targets of *PtoeIF5A1* using approaches such as RNA immunoprecipitation sequencing (RIP-seq) or ribosome profiling will be essential to dissect its regulatory network. Moreover, elucidation of how its hypusination status dynamically modulates translation elongation activity in response to fluctuating internal and external stimuli will provide mechanistic insights into its pleiotropic functions. These endeavors will advance the understanding of translational regulatory mechanisms governing stress adaptation in trees and provide a theoretical foundation for devising genetic improvement strategies aimed at enhancing stress resilience in woody plants.

## 5. Conclusions

In this study, we systematically identified four *eIF5A* genes in *P. tomentosa* and functionally characterized the pleiotropic roles of PtoeIF5A1 in developmental regulation, programmed cell death, and stress responses. *PtoeIF5A1* exhibited a distinct tissue-specific expression pattern, with predominant accumulation in the roots and secondary xylem, which are tissue characterized by canonical PCD and secondary wall thickening during maturation. Heterologous overexpression of *PtoeIF5A1* in *Arabidopsis* promoted bolting and stem elongation, culminating in early flowering, while its transient expression in tobacco leaves induced PCD and premature senescence. Furthermore, *PtoeIF5A1* is strongly and persistently upregulated by salt stress, and its overexpression in *Arabidopsis* confers enhanced salt tolerance as evidenced by increased fresh weight under NaCl treatment. These findings establish PtoeIF5A1 as a positive regulator of development, PCD, and salt tolerance, which integrates developmental signals, stress cues, and senescence at the translational level, providing valuable genetic resources for the molecular breeding of stress-resilient trees.

## Figures and Tables

**Table 1:** Various Features of *eIF5A* Genes in *P. tomentosa*.

Gene Name	ORF/bp	Protein/aa	Molecular Weight	Theoretical pl	Instability Index	Aliphatic Index	Grand Average of Hydropathicity
*PtoelF5A1*	480	160	39,664.37	5.21	43.60	27.08	0.746
*PtoelF5A2*	483	161	39,797.22	5.23	36.97	27.95	0.715
*PtoelF5A3*	480	160	39,632.25	5.21	43.27	26.67	0.742
*PtoelF5A4*	483	161	39,675.02	5.23	39.14	28.16	0.708

## Data Availability

The original contributions presented in this study are included in the article/Supplementary Materials. Further inquiries can be directed to the corresponding authors.

## Supplementary Materials

The following supporting information can be downloaded at: https://www.mdpi.com/article/10.3390/plants15172702/s1, Table S1. Gene-specific primers used in this study. Table S2. eIF5A genes from *Populus tomentosa* and other species used in this study. Figure S1. Physiological responses of WT and transgenic lines under salt stress. WT, *PtoeIF5A1* OE-1, *PtoeIF5A1* OE-2, and *PtoeIF5A4* transgenic seedlings were cultured on 0.5 × MS medium supplemented with 0, 100, or 150 mM NaCl for 15 days. (A) Relative ion leakage (RIL) was measured as an indicator of membrane damage. (B) Free proline content was determined as a marker of osmotic adjustment. Three independent measurements were performed. Data are presented as the mean ± SD from three replicates. Statistical significance was determined by Student’s *t*-test. Asterisk (*) indicates a difference at the *p* < 0.05 level. Asterisks (**) in each column indicate a significant difference at the *p* < 0.01 level. Figure S2. Flowering time analysis of WT and transgenic lines. WT, *PtoeIF5A1* OE-1, and *PtoeIF5A1* OE-2 transgenic seedlings were grown under long-day conditions. (A) Days to flowering. (B) Rosette leaf number at the flowering stage. Three independent measurements were performed. Data are presented as the mean ± SD from three replicates. Statistical significance was determined by Student’s *t*-test. Asterisk (*) indicates a difference at the *p* < 0.05 level. Asterisks (**) in each column indicate a significant difference at the *p* < 0.01 level. Figure S3. Relative expression levels of *PtoeIF5A* family genes under different abiotic stress treatments for 24 h, normalized to *PtoAction9*. The transcript abundances of *PtoeIF5A* family members in *P. tomentosa* were examined by RT-qPCR following exposure to high light, alkali, drought, heat, cool, and salt stresses for 24 h. *PtoAction9* was used as the internal reference gene, and its expression level was set to 1. Three independent measurements were performed. Data are presented as the mean ± SD from three replicates. Statistical significance was determined by Student’s *t*-test. Asterisk (*) indicates a difference at the *p* < 0.05 level. Asterisks (**) in each column indicate a significant difference at the *p* < 0.01 level.
